# The neuroprotective role of melatonin in neurological disorders

**DOI:** 10.1002/jnr.24220

**Published:** 2018-03-01

**Authors:** B. S. Alghamdi

**Affiliations:** ^1^ Department of Physiology, Faculty of Medicine King Abdulaziz University Jeddah KSA; ^2^ Neuroscience Unit, Faculty of Medicine King Abdulaziz University Jeddah KSA

**Keywords:** melatonin, ischemia, alzheimer, parkinson

## Abstract

Melatonin is a neurohormone secreted from the pineal gland and has a wide‐ranging regulatory and neuroprotective role. It has been reported that melatonin level is disturbed in some neurological conditions such as stroke, Alzheimer's disease, and Parkinson's disease, which indicates its involvement in the pathophysiology of these diseases. Its properties qualify it to be a promising potential therapeutic neuroprotective agent, with no side effects, for some neurological disorders. This review discusses and localizes the effect of melatonin in the pathophysiology of some diseases.

## 1 INTRODUCTION

Melatonin is a neurohormone secreted from the pineal gland, which is situated at the center of the brain (Tan, Manchester, Sanchez‐Barcelo, Mediavilla, & Reiter, 2010). It has a wide range of regulatory and protective effects, such as synchronizing circadian rhythm, protecting against oxidative stress, regulating energy metabolism, modulating the immune system, and postponing the ageing process (Tan, Manchester, Terron, Flores, & Reiter, 2007; De Pedro, Martinez‐Alvarez, & Delgado, 2008; Caballero et al., 2009; Shieh, Wu, Cheng, & Cheng, J., 2009; Hardeland, 2010; Tan et al., 2010). Melatonin is also secreted from extra‐pineal sources, the highest melatonin release coming from the gut and the skin (Tan et al., 2007). Other extra‐pineal sources are the retina, the testes, the ovary, the placenta, glial cells, and lymphocytes (Tan et al., 2007). However, melatonin secreted from extra‐pineal sources has a small effect on the plasma melatonin circadian oscillation, as pinealectomy is found to disturb melatonin rhythm (Pelham, 1975). Extra‐pineal areas secrete melatonin, which remains within these tissues and acts mainly as an antioxidant agent (Tan et al., 2007).

Normally, melatonin is secreted during the first year of life, starting from a very low level before the age of three months and then increasing rhythmically to reach its peak at 1–3 years, followed by a gradual decrease until adulthood (Waldhauser, Ehrhart, & Forster, 1993). Diurnally, melatonin is found to be highly secreted between 03:00 and 04:00 (Claustrat & Leston, 2015). Melatonin circulating in the blood is distributed widely throughout the body, including in saliva, urine, cerebrospinal fluid, preovulatory follicles, semen, amniotic fluid and milk (Reiter, 1991). The melatonin level in the bloodstream indicates active pineal gland function (Reiter, 1991). Melatonin is lipophilic and hydrophilic in nature, and this amphiphilic property bestows the advantage of crossing the blood–brain barrier (Pardridge & Mietus, 1980).

## 2 BIOSYNTHESIS

Melatonin synthesis occurs in pinealocytes from tryptophan, which is converted to serotonin and finally into melatonin in a four‐step process (Tan et al., 2010). Tryptophan is naturally present in the bloodstream and its acute depletion has been shown to be capable of reducing the nocturnal melatonin level in humans (Zimmermann et al., 1993). As folate is essential in the methylation step of converting N‐acetylserotonin to melatonin, folate deficiency decreases melatonin release in rats (Fournier, Ploye, Cottet‐Emard, Brun, & Claustrat, 2002). Moreover, vitamin B6 (pyridoxine) plays an important role in tryptophan decarboxylation and increases the release of melatonin by the pineal gland in children but not in adults (Munoz‐Hoyos et al., 1996; Luboshitzky et al., 2002).

Two enzymes are essential for the conversion of serotonin into melatonin, namely serotonin‐N‐acetyltransferase (NAT; also known as arylalkylamine N‐acetyltransferase [AANAT] and hydroxyindole‐O‐methyltransferase [HIOMT]; [Tan, Manchester et al., 2010]). It has been found that the expression of mRNAs encoding these enzymes in a chicken's pineal gland is controlled by day/night circadian oscillators that reach their maximum level at night (Bernard et al., 1999). Norepinephrine (NE) has a key role in initiating melatonin synthesis, in which the binding of NE to adrenergic 1 receptors in pinealocytes stimulates cyclic AMP (cAMP) and therefore NAT formation (Tan et al., 2010).

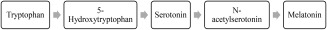

## 3 METABOLISM

In the brain, melatonin is oxidized by formamidase and produces N1‐acetyl‐N2‐formyl‐5‐methoxykynuramine (AFMK; Hirata, Hayaishi, Tokuyama, & Seno, 1974; Cardinali, 1981). N1‐acetyl‐5‐methoxy‐kynuramine (AMK) is another well‐known melatonin metabolite. AFMK and AMK can be produced by enzymatic, free radical or ultraviolet radiation pathways (Hirata et al., 1974; Cardinali, 1981; Tan et al., 2001; Hardeland, Tan, & Reiter, 2009). Both melatonin metabolites are considered potent antioxidants with the capacity to scavenge free radicals and activate the free radical scavenging cascade (Tan et al., 2001; Hardeland et al., 2009).

In the liver, circulating melatonin is hydroxylated by cytochrome CYP1A2 into 6‐hydroxymelatonin, with wide variance in CYP1A2 activity between individuals (Gunes & Dahl, 2008). Different factors are shown to affect this activity, which in turn alters the melatonin level. For example, caffeine is shown to counteract CYP1A2 action, thus inhibiting melatonin metabolism in the liver and raising the melatonin level (Hartter et al., 2003; Hartter et al., 2006). Cigarette smoking is shown to activate CYP1A2 and depress the exogenous melatonin level (Ursing, von Bahr, Brismar, & Rojdmark, 2005). 6‐hydroxymelatonin is excreted in the urine in the form of sulphate and glucuronide (Isidorov & Nazaruk, 2017). The urinary level of 6‐hydroxymelatonin sulphate is reportedly associated with the plasma melatonin level (Arendt, Bojkowski, Franey, Wright, & Marks, 1985).

## 4 REGULATION OF MELATONIN SECRETION

Melatonin in mammals is released in a rhythmic oscillation pattern, which is regulated by suprachiasmatic nuclei (SCN) of the hypothalamus in animals and humans (Cohen & Albers, 1991; Moore, 1992; Edgar, Dement, & Fuller, 1993; Cardinali & Pevet, 1998). Melatonin release is controlled by the diurnal cycle, in which daylight supresses melatonin release by signals transmitted by the retino‐hypothalamic tract to the SCN. It has been found that an adequate intensity of artificial light in the room at night suppresses melatonin release in humans (Lewy, Wehr, Goodwin, Newsome, & Markey, 1980). The intensity of light is inversely proportional to the nocturnal plasma level of melatonin (Bojkowski et al., 1987). Exposure to a light intensity of about 2000 to 2500 lx between 02:00 and 04:00 significantly supresses the melatonin plasma level (Bojkowsk et al., 1987).

A neuronal connection is found between the SCN and the sympathetic system through the superior cervical ganglion, in which fibres projected from the ganglion directly synapse with the pineal gland and secrete norepinephrine to activate melatonin synthesis and release (Moller, 1992; Moller & Baeres, 2002). Therefore, blocking 1‐adrenergic receptors by atenolol suppresses melatonin release, as detected by urinary melatonin metabolites (Arendt et al., 1985).

## 5 SOURCES

A wide variety of dietary elements contain melatonin, including nuts, seeds, fruits, vegetables, and cereals (Manchester et al., 2000; Iriti, 2009; Iriti & Faoro, 2009; Paredes, Korkmaz, Manchester, Tan, & Reiter, 2009). A radioimmunoassay showed an increased level of plasma melatonin following an intake of food rich in melatonin (Hattori et al., 1995). It is known that the plasma level of melatonin is reflected in melatonin level in the body (Tan et al., 2010). The normal melatonin level in the blood varies diurnally within a range of a few pg/ml during the day to more than 50 pg/ml at night. However, the plasma melatonin level is much lower than in other areas such as the gut and bone marrow, both of which are considered extra‐pineal sources of melatonin. Thus, the level of melatonin in plasma does not reflect its concentration in other areas of the body (Huether, 1993; Tan et al., 1999).

## 6 MELATONIN IN THE CENTRAL NERVOUS SYSTEM (CNS)

AANAT and HIOMT are two crucial enzymes in the melatonin synthesis pathway. AANAT is important in converting serotonin into N‐acetylserotonin, while HIOMT converts N‐acetylserotonin into melatonin (Hirata et al., 1974). The HIOMT enzyme plays a physiological role in regulating melatonin peak release at night (Ribelayga, Pevet, & Simonneaux, 2000).

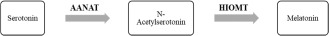

Previously, it was believed that the pineal source of melatonin is the origin of the level of melatonin in the blood and CNS. However, recent data show that there are other CNS sources of melatonin. Reverse transcription polymerase chain reaction (RT‐PCR) identified mRNA encoding AANAT and HIOMT in a wide range of rat brain areas (Stefulj et al., 2001). This reflects the possible endogenous melatonin synthesis and release from different brain regions. AANAT and HIOMT enzymes are found in astrocytes, which release melatonin in cell cultures of the rat cortex and glioma C6 cell line (Liu et al., 2007).

Both melatonin metabolites, AFMK, and AMK are present in the brain. AFMK was first discovered in a rat brain in 1974 by Hirata et al. and since then it is believed to be the main melatonin metabolite in the brain (Hirata et al., 1974; Tan et al., 2007).

The AFMK concentration in cases of meningitis is found to be several times higher than in normal healthy cerebrospinal fluid (CSF; Silva, Ximenes, Livramento, Catalani, & Campa, 2005). Given that every unit of melatonin produces a single AFMK, a high concentration of CSF AFMK in meningitis cases exceeds the pineal gland's capacity to produce melatonin, which in turn suggests another source of melatonin secretion to the CSF. The synthesis and release of melatonin are considered to be a stress‐mediated mechanism in which melatonin is a potent neuroprotector and antioxidant (Carloni et al., 2008; Carretero et al., 2009; Manda, Ueno, & Anzai, 2009). Stress‐mediated melatonin release has been demonstrated in the case of acute pancreatitis in rats and severe traumatic brain in humans (Jaworek et al., 2003; Seifman et al., 2008).

## 7 THE PINEAL GLAND AND MELATONIN IN THE CSF

The direct connection of the pineal recess to the third ventricle was first recognized in 1969 (Sheridan, Reiter, & Jacobs, 1969; Sheridan & Reiter, 1970). Melatonin concentration in sheep was found to reach its highest level in the third ventricle near the pineal recess, the concentration gradually decreasing in the CSF with increased distance from the third ventricle (Tricoire, Locatelli, Chemineau, & Malpaux, 2002). The high‐performance liquid chromatography (HPLC) technique used in humans showed a high level of melatonin in the third ventricles and lower levels in the lateral and fourth ventricles (Longatti et al., 2007). However, HPLC can measure the level of free melatonin only, thus missing a high percentage of melatonin in bound form (Rizzo et al., 2002). Surgical sealing of the pineal recess leads to a drop in the melatonin concentration in the third ventricle (Tricoire et al., 2002). This result is consistent with the direct release of pineal melatonin to third ventricle through the pineal recess. All of these data have opened the field for more research to investigate whether the CNS level of melatonin is of pineal origin only. What is the role of melatonin secreted from the glial cells? Is it activated during stress only or is there a regulatory system controlling its synthesis and release?

## 8 DISTRIBUTION OF MELATONIN RECEPTORS IN THE NERVOUS SYSTEM

Melatonin binds to two types of G‐protein‐coupled receptors, namely MT1 and MT2 (Dubocovich & Markowska, 2005; Benitez‐King, 2006; Ng, Leong, Liang, & Paxinos, 2017). MT1 is distributed in a wide area of the nervous system, including the hippocampus, the caudate putamen, the suprachiasmatic nucleus (SCN), the paraventricular nucleus, the periventricular nucleus, the supraoptic nucleus, the Meynert nucleus, the nucleus accumbens, the substantia nigra tuberomammillary nucleus, the mammillary bodies, and the retina (Dubocovich & Markowska, 2005; Wu et al., 2006; Ng et al., 2017). On the other hand, MT2 is mainly expressed in the hippocampus, the SCN and the retina (Dubocovich & Markowska 2005; Ng et al., 2017). Both receptors are expressed by neurons and glial cells of the cerebral and cerebellar cortex, thalamus, and pineal gland (Brunner et al., 2006; Wu et al., 2006; Ng et al., 2017). Interestingly, it has been found that expression of the mRNA encoding MT1 receptor is affected by the day/night cycle, and that there is a relation between plasma melatonin level and MR1 mRNA expression (Masana, Benloucif, & Dubocovich, 2000).

## 9 THE BRAIN IS AN ORGAN THAT IS SENSITIVE TO ENERGY DISTURBANCE

Although the brain makes up only 2% of the human body weight, it consumes around 20% of the body's oxygen. This high level of oxygen consumption can initiate a harmful process known as oxidative stress. Oxidative stress is the appearance of reactive oxygen species (ROS) in a way that exceeds the capacity of the antioxidant effect (Gupta Y., Gupta, & Kohli, 2003). ROS are unstable molecules with one or more unpaired electrons in their outer layer (Guetens, De Boeck, Highley, van Oosterom, & De Bruijn, 2002); for example, superoxide (O_2_^−^), hydroxyl (OH^−^), peroxyl (RO_2_^−^), alkoxyl (RO^−^) radicals or covalent molecules as H_2_O_2_ (Caimi et al., 2004). These molecules are harmful and destroy DNA, proteins, and the cell membrane (Gupta et al., 2003).

A high level of fat in the brain distributed in the cell membranes and myelin sheath makes it more prone to targeted damage by ROS. A lower level of antioxidant enzymes compared to other body areas generates an imbalance between ROS production and the opposing effect of antioxidants (Skaper et al., 1999). ROS damage extensively affecting the brain function leaves the blood–brain barrier leaky, disturbing mitochondrial respiration and changing the tubulin arrangement (Gupta et al., 2003). Studies showed that ROS enhances a release of excitatory neurotransmitter as glutamate to the extracellular space, which acts on different types of receptors, mainly NMDA receptors, and triggers an anoxic depolarization (Gilman, Bonner, & Pellmar, 1993). Moreover, ROS alters gene expression, mediates apoptotic cascade, and eventually decreases neuronal viability (Gilgun‐Sherki, Rosenbaum, Melamed, & Offen, 2002).

## 10 NEUROLOGICAL CONDITIONS AND MELATONIN

### 10.1 Melatonin and ischaemia

Ischaemic stroke is the second main cause of death and the leading cause of disability worldwide (Flynn, MacWalter, & Doney, 2008; Mathers, Boerma, & Ma Fat, 2009). There are two types of stroke, ischaemic stroke, which represents 85% of all strokes, and high‐mortality haemorrhagic stroke, which accounts for 15% of all strokes (Flynn et al., 2008). A complex cascade of cellular injury events is set in motion during ischaemia, consisting of excitotoxicity, ROS production, and inflammation.

Neurons are very active excitable cells that need a high metabolic rate to maintain their energy‐dependent activities (Hossmann, 1994). Thus, any restriction in the cerebral blood flow, as in the case of ischaemic stroke, is considered a serious situation for neurons, as they need a steady and consistent supply of oxygen and glucose. In the case of oxygen glucose deprivation, cell viability is affected by different mechanisms, starting from energy deprivation, which affects one of the main ATP‐dependent pumps, Na^+^/K^+^‐ATPase, causing its failure and reversing its function (efflux of K^+^ and influx of Na^+^; Stys, 1998). A high level of intracellular Na^+^ [Na^+^]_I_ initiates anoxic depolarization, activates voltage gated calcium channels (VGCC) and reverses Na^+^‐Ca^2+^ exchange (Stys, 1998). Consequently, Ca2+ ions move inside the cells and activate a Ca^2+^ mediated injury process (Stys, 1998). This is consistent with the findings of Muller and Ballanyi, in which ischaemia initiated anoxic depolarization coincident with a huge increase in intracellular Ca^2+^ [Ca^2+^]_I_ (Muller & Ballanyi, 2003). The level of glutamate, a major excitatory neurotransmitter, during ischaemia is increased extracellularly through inverse of its uptake and the release from presynaptic neurons (Obrenovitch, 1996; Rossi, Oshima, & Attwell, 2000). A prolonged high level of glutamate binds to NMDA and non‐NMDA receptors and initiates an excitotoxicity cell injury (Rothman & Olney, 1986; Arundine & Tymianski, 2003). NMDA receptors mediate Ca^2+^ influx and amplify Ca^2+^ overload, while AMPA receptors allow Na^+^ entry, leading to cell swelling and brain oedema (Dirnagl, Iadecola, & Moskowitz, 1999; Lipton, 1999).

Excitotoxicity is followed by an oxidative stress neurotoxic effect. High [Ca^2+^]_I_ overload activates multiple oxidative enzymes (e.g., phospholipases, cyclooxygenases, NO synthase, and proteolytic enzymes). These enzymes accelerate the formation of free radicals, which in turn mediates a series of cellular injury events such as lipid peroxidation, DNA damage, mitochondrial injury, and blood–brain barrier (BBB) breakdown, which mediates brain oedema (Dirnagl et al., 1999; Lipton, 1999, Rodrigo, Fernandez, Serrano, Peinado, & Martinez, 2005).

Excitotoxicity, a high [Ca^2+^]_I_ level and oxidative stress are followed by inflammation. The inflammation stage is started by the activation of different factors such as NFκB, hypoxia‐mediated factor 1, and STAT3, which are responsible for the production of inflammatory cytokines (TNF‐α, IL‐1β), enzymes (iNOS, COX‐2), and adhesion molecules (ICAM‐1, selectins) and for increasing the number of activated phagocytes (Dirnagl et al., 1999; Iadecola & Alexander, 2001).

These stages of ischaemic injury start from the first minutes of ischaemic insult and persist for several days, in which the ischaemic damage started at the core of the injury spreads out to the penumbra, a hypoperfused and functionally disturbed, but viable tissue (Dirnagl et al., 1999).

Clinically, in acute ischaemic stroke, thrombolysis or thrombectomy are established to remove the obstruction in the blood vessels and thereby regain cerebral circulation in the affected area. However, reperfusion will play a role in increasing the production of oxygen free radicals and therefore exacerbate oxidative stress and inflammatory injuries (Chen et al., 2011). Therefore, combining neuroprotective strategies with thrombosis or thrombolysis provides an effective way of treating stroke patients with better outcomes. The main objective is to save the penumbra from cell death.

**Figure 1:**
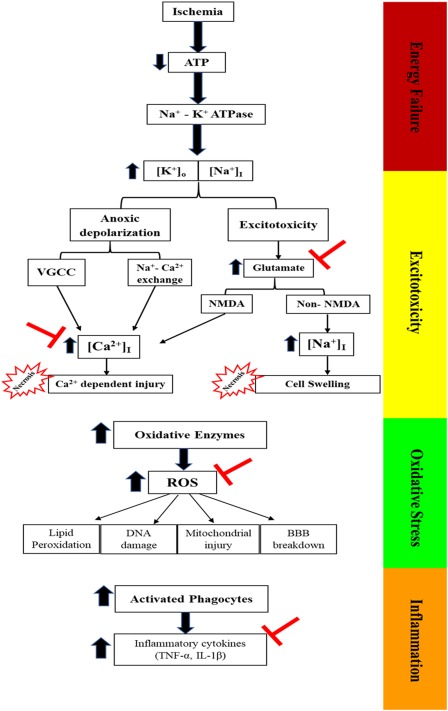
Ischaemia pathophysiology at four stages: energy failure, excitotoxicity, oxidative stress and inflammation. (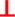) Represents the site of melatonin action

Melatonin is considered one of the most potent antioxidant, playing an important protective role in ischaemic injury (see Figure 1; Watson, Diamandis, Gonzales‐Portillo, Reyes, & Borlongan, 2016; Wu et al., 2017). In middle cerebral artery occlusion (MCAO), a model of acute ischaemia in rats, receiving pineal gland transplantation improves the motor outcome and decreases the infarct size through the secretion of melatonin (Borlongan et al., 2003). Exogenous melatonin administration (4 mg/kg) significantly improves motor outcome and decreases infarct size by 40% in pinealectomized rats subjected to MCAO (Kilic, Ozdemir, Bolay, Kelestimur, & Dalkara, 1999). Moreover, it has been found that melatonin injections protect against oxidative brain injury in cases of subarachnoid haemorrhage (SAH) in rat models (Martinez‐Cruz, Espinar, Pozo, Osuna, & Guerrero, 2002; Ersahin et al., 2009; Wu et al., 2017).

Melatonin has a role in maintaining Ca^2+^ homeostasis and preventing any impairment. Moreover, it has been shown to decrease the extracellular level of glutamate in hippocampal sections by reversing its release in an oxygen glucose deprivation (OGD) model of rat ischaemia (Patino et al., 2016). Melatonin prevents an acid‐induced increase in [Ca^2+^]_I_ levels (Bhattacharya, Pandey, Paul, & Patnaik, 2014). In addition, it diminishes a glutamate‐dependent increase in [Ca^2+^]_I_ level by decreasing parvalbumin and hippocalcin, calcium‐buffering proteins in the rat's cerebral cortex (Koh, 2012).

A fluorescence live‐animal imaging system has shown that melatonin reduces free radical generation by acting on the MT2 receptor after a transient middle cerebral artery ischaemia in mice (Chern et al., 2012). Moreover, melatonin reportedly reduces Nox2 and Nox4 expression, thus decreasing the oxidative stress damage that is seen in ischaemic/reperfusion injury in rats (Li et al., 2014). Melatonin administration 60 minutes after MCAO has been shown to play a role as an antioxidant in reducing nitrite and malondialdehyde (MDA) levels and improving motor behavioural outcomes and brain oedema (Bhattacharya et al., 2014).

A study conducted on 45 human newborns diagnosed by hypoxic‐ischaemic encephalopathy showed that melatonin combined with hypothermia decreases plasma‐free radicals (nitric oxide [NO]), seizure attacks, and white matter insults and finally improves neurological outcomes (Aly et al., 2015). Another 10 asphyxiated human newborns treated with 8 doses of 10 mg melatonin demonstrated a better antioxidant effect, reducing serum malondialdehyde and nitrite/nitrate levels, therefore improving their survival outcomes (Fulia et al., 2001).

Melatonin administration decreases macrophage brain infiltration in transient focal cerebral ischaemia and MCAO of rats, which in turn prevents excess secretion of inflammatory cytokines and subsequent inflammatory injury (Chen et al., 2006; Lee et al., 2007). RT‐PCR demonstrated that melatonin administration significantly reduced the expression of interleukin‐1 beta (IL‐1β) and tumour necrosis factor alpha (TNF‐α) in ageing MCAO rats (Paredes et al., 2015). Messenger RNA expression of Bcl‐2‐associated death promoter (BAD), Bcl‐2‐associated X protein (BAX), glial fibrillary acidic protein (GFAP), B‐cell lymphoma 2 (Bcl‐2), and sirtuin 1 has been measured by reverse‐polymerase chain reaction. This also preserves the integrity of BBB and significantly diminishes its dysfunction through different mechanisms such as NO, ROS, and conserves tight junctions (Chen et al., 2006; Grossetete, Phelps, Arko, Yonas, & Rosenberg, 2009; Song et al., 2014; Moretti et al., 2015; Alluri et al., 2016). All these results suggest that melatonin can be of important therapeutic value in preventing BBB leakage and brain oedema.

### 10.2 Melatonin and Alzheimer's disease (AD)

Alzheimer's disease is an age‐related neurodegenerative disease marked by toxic protein aggregation inside and outside the neurons. Extracellular β‐amyloid (Aβ) and intracellular neurofibrillary tangles (NFTs) are the hallmark of Alzheimer's disease (Ittner & Gotz, 2011). These abnormal proteins are found to be accumulated in memory‐related areas in the brain, such as the neocortex and hippocampus, leading to progressive decline in cognitive function (He, Dong, & Huang, 2010). The accumulation of toxic proteins mediates oxidative stress, synaptic dysfunction and neuronal loss (Sultana & Butterfield, 2010; Jeong, 2017). The aetiology of the disease is still not clear; however, several factors are found to contribute to the disease, such as genetic factors, sex, lipid metabolism, ageing, diet, and metal ion toxicity (Sultana & Butterfield, 2010; Leszek, Sochocka, & Gasiorowski, 2012; Mustapic et al., 2012). The most common genes attributed to Alzheimer's disease are the amyloid precursor protein (APP), apolipoprotein E (ApoE), and presenilins 1 (PS1) and 2 (PS2; Price & Sisodia, 1998; Shimohama, 2000; Thomas, Thomas C, Radcliffe, & Itsiopoulos, 2015; Dominguez & Barbagallo, 2016; Loffler, Flunkert, Temmel, & Hutter‐Paier, 2016; Canerina‐Amaro et al., 2017; Dong, Gim, Yeo, & Kim, 2017; Li et al., 2017).

High Aβ production is considered the primary cause of neuropathology in AD (Hardy & Selkoe, 2002). Senile plaques consist of Aβ peptides that are made up of around 40–43 amino acids (Soto, Branes, Alvarez, & Inestrosa, 1994; Soto, Castano, Frangione, & Inestrosa, 1995; Selkoe, 1998). Amyloid protein precursor (APP) undergoes proteolytic cleavage by β‐secretase at the C‐terminal and γ‐secretase at the N‐terminal to produce Aβ peptide (Nunan & Small, 2000). On the other hand, α‐secretase splits APP at the middle region of the Aβ sequence and does not produce Aβ (Vardy, Catto, & Hooper, 2005). Different gene mutations are suggested to play a role in Aβ formation, such as PS1 and PS2 (Bruni, 1998; Liu, Zhou, van Heerikhuize, Hofman, & Swaab, 1999; Seiffert et al., 2000; Lleo, Berezovska, Growdon, & Hyman, 2004). These gene mutations mediate soluble Aβ production. However, aggregation and deposition of soluble Aβ peptides produces a toxic fibrillary Aβ (Yankner, 1996; Alvarez et al., 1998; Soto et al., 1998). Two common types of Aβ are found in brain, Aβ40 and Aβ42, which differ in the number of amino acids (40 and 42, respectively) and are similar in their hydrophobic property (He et al., 2010). This property mediates Aβ fibrils to aggregate in a β‐sheet structure known as amyloid plaques (He et al., 2010). A cascade of Aβ plaque formation was presented by Louise C. Serpell, in which the cascade started to form APP, soluble Aβ, Aβ oligomers, Aβ protofilament, Aβ fibrils, and Aβ plaques (Serpell, 2000). Aβ fibrils are found to be condensed in the AD brain, and several studies reported its neurotoxic effects, including synaptic dysfunction, neuronal death, and hence, dementia (Lorenzo & Yankner, 1994; Younkin, 1995; Forloni, 1996; Selkoe, 1999; Iwata et al., 2000; Puglielli, Tanzi, & Kovacs, 2003).

Oxidative stress is involved in the aetiology and the subsequent neurodegenerative pathology of AD (Baldeiras et al., 2008; Greilberger et al., 2008; Padurariu et al., 2010; Ferreiro et al., 2012). A high level of free radicals in AD is mediated by several causes, such as Aβ deposition, mitochondrial dysfunction, inflammation, and activated microglia (Padurariu, et al., 2013). Aβ plaques are considered one of the main causes of oxidative stress in AD, with a two‐way effect, in which oxidative stress mediates lysosomal production of Aβ and Aβ itself induces lysosomal membrane dysfunction and finally cell death (Zheng, Roberg, Jerhammar, Marcusson & Terman, 2006).

**Figure 2:**
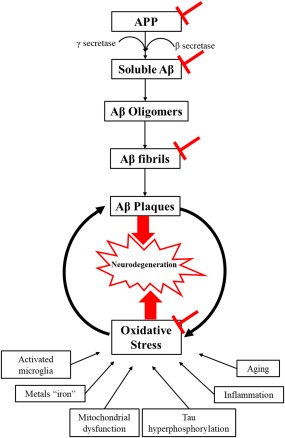
Alzheimer's pathophysiology with melatonin site of action (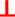)

The antiamyloidogenic effect of melatonin in AD has been reported in various studies (Figure 2; Shukla, Govitrapong, Boontem, Reiter, & Satayavivad 2017). It has been shown that melatonin inhibits the formation of soluble APP in vitro, which in turn could prevent Aβ production (Lahiri, 1999), consistent with the decrease in APP mRNA level after melatonin administration in P12 cells, but not in human neuroblastoma cells (Song & Lahiri, 1997). Moreover, it has been reported that melatonin can interfere with Aβ fibril production in vitro through interacting with A 40 and A 42 (Pappolla et al., 1998; Pappolla et al., 2000; Poeggeler et al., 2001).

It has been found that long‐term application of melatonin (for around two months) reduced immunoreactive Aβ deposition in the hippocampus and cortex by 43% and 37%, respectively in a transgenic rat model of Alzheimer's (Olcese et al., 2009). Melatonin administration during the active stage of disease progression reduced amyloid deposition in the hippocampus (β1–42 and β1–40) and frontal cortex (β1–42), decreased degenerative changes in the hippocampus, prevented mitochondrial dysfunction, and delayed anxiety and cognitive impairment in a sporadic rat model of Alzheimer's (Rudnitskaya, Muraleva et al., 2015). Chronic melatonin application after intracerebroventricular Aβ1–42‐injection demonstrated diminished tau hyperphosphorylation and Aβ mediated memory deficits, thus avoiding neurodegeneration in mice hippocampus (Ali & Kim, 2015).

Melatonin is also reported to be a potent antioxidant in AD (Figure 2; Shukla et al., 2017). Several studies have found that melatonin reduces Aβ mediated oxidative stress and lipid peroxidase (Daniels, van Rensburg, van Zyl, & Taljaard, 1998; Srinivasan et al., 2005; Shukla et al., 2017). Melatonin has been shown to regulate the level of mRNA encoding in some antioxidant enzymes (SOD‐1, glutathione peroxidase, and catalase) to its normal level in the cortex of transgenic AD mice (Olcese et al., 2009). It reduced the production of ROS by inhibiting the formation of NADPH oxidase, the main source of oxidative stress in AD, in β‐stimulated microglial culture (Zhou, Zhang, Zhao, & Wei, 2008). The oxidative stress end products are abundantly found in AD brains. Lipid peroxidation, DNA damage and oxidative altered protein metabolites are routinely identified in postmortem AD brains (Subbarao, Richardson, & Ang, 1990; Mecocci, MacGarvey, & Beal, 1994; Smith, Sayre, Monnier, & Perry, 1995; Markesbery, 1997). Moreover, neuroinflammation and the activated microglia worsen oxidative stress by enhancing NO generation, which mediates neuronal degeneration, especially in proximal death (Weldon et al., 1998).

It has been found that long administration of oral melatonin enhances hippocampal synaptic growth and preserves the neuronal and glial structure in a sporadic rat model with Alzheimer's (Stefanova et al., 2015). In addition, melatonin reportedly improves spatial memory, reduces synaptic dysfunction, and decreases astrogliosis in the rat hippocampus after intracerebroventricular injections of soluble Aβ1–42 (Zhang, Wang, Ren, Hu, & Bi, 2016). Further research demonstrated that melatonin diminishes apoptotic mediators in a senescence‐accelerated mice (SAMP8) model of Alzheimer's (Gutierrez‐Cuesta et al., 2008).

Clinically, multiple studies reported a decrease in melatonin level in AD patients compared to healthy people (Skene et al., 1990; Uchida, Okamoto, Ohara, & Morita, 1996; Liu et al., 1999; Mishima et al., 1999; Ohashi et al., 1999). The melatonin level in postmortem ventricular CSF is adversely related to Braak and modified Braak staging in the human cortex (Zhou, Liu, Kamphorst, Hofman, & Swaab, 2003). Thus, the melatonin level is considered as a marker for the progression of AD neuropathology. In Braak staging I and II “pre‐clinical” stages of Alzheimer's disease, melatonin circadian oscillation is lost owing to noradrenergic dysfunction and monoamine oxidase generation (Wu et al., 2003). A retrospective study showed that melatonin significantly improved Beck depression inventory scores (BDI), mini‐mental state examination results (MMSE), the cognitive subscale of the Alzheimer's disease assessment scale (ADAS‐Cog) and sleep quality in mildly cognitively impaired patients (Cardinali, Vigo, Olivar, Vidal, Furio, & Brusco, 2012). However, more studies are needed to investigate the mechanism and the therapeutic potentials of melatonin in AD patients.

Melatonin is known as anti‐β amyloid aggregation, antioxidant and anti‐inflammatory and therefore prevents synaptic dysfunction, neuronal loss and cognitive impairment.

### 10.3 Melatonin and Parkinson's disease (PD)

Several million people suffer from PD worldwide (Elbaz & Moisan, 2008; Wirdefeldt, Adami, Cole, Trichopoulos, & Mandel, 2011). Several risk factors are positively associated with PD incidence, including genetic factors, age, exposure to lead and manganese, and consumption of dairy products; while coffee and tea are inversely associated with PD (Elbaz & Moisan, 2008; Wirdefeldt et al., 2011; Hughes et al., 2017). A lot of controversies have been raised in the way of association of cigarette smoking with PD (Ma, Liu, Neumann, & Gao, 2017).

PD is characterized by dopaminergic neuronal loss in the substantia nigra pars compacta (SNc), leading to depletion in striatal dopamine, which in turn affects smooth, coordinated motor movements, mediating the appearance of rigidity, tremor, bradykinesia, and postural instability (Zhang et al., 1999; Tansey, McCoy, & Frank‐Cannon, 2007; Maguire‐Zeiss & Federoff, 2010). Non‐motor symptoms have also been reported in PD patients, including impulse control disorders (ICDs) and neuropsychiatric, autonomic, sleep, and sensory dysfunction (Weintraub, Comella, & Horn, 2008; Garcia‐Ruiz, Chaudhuri, & Martinez‐Martin, 2014). The pathological hallmark of PD is dopaminergic neuronal death, which may affect 60% of total dopaminergic neurons, thus affecting the connections with other neurons (Zigmond MJ). The histological hallmark of PD is the distribution of Lewy bodies and α‐synuclein protein aggregation on neurons (Spillantini, Crowther, Jakes, Hasegawa, & Goedert, 1998; Shults, 2006). Aggregation of Lewy bodies compromises mitochondrial dynamics, which mediates ROS release and cell death (Pozo Devoto, & Falzone, 2017).

Several types of PD animal models have been established to mediate dopamine neuronal death and augment the generation of sensory and motor deficit, which in turn expose PD symptoms, such as tremor, rigidity and akinesia (Schober, 2004; Terzioglu & Galter, 2008). Two mechanisms of PD animal model induction are commonly used, namely nigrostriatal injection of 6‐hydroxydopamine (6‐OHDA) and cerebral injections of neurotoxins such as MPTP (Schober, 2004; Terzioglu, & Galter, 2008). Transgenic models of PD have also been established, but some studies reflect some concerns about transgenic models and neurotoxic induced models, which should be taken into consideration (Terzioglu & Galter, 2008).

**Figure 3:**
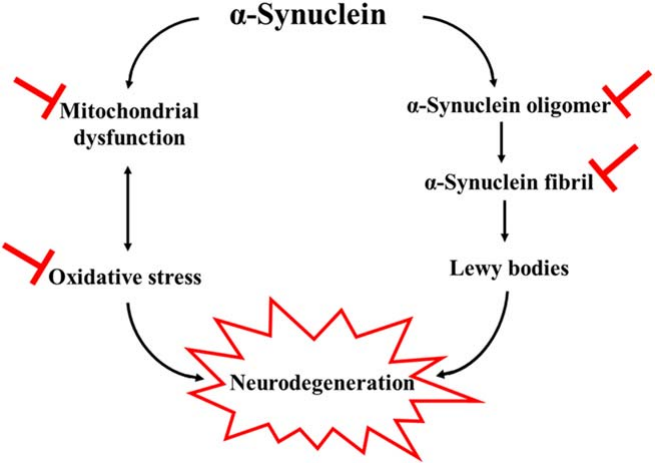
Parkinson's disease pathophysiology with melatonin site of action (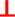)

Evidence has been accumulating that age‐associated PD is combined with oxidative stress (Olanow 1992, Padurariu, Ciobica et al., 2013). It has been reported that melatonin injections interfere with lipid peroxidation in the hippocampus and striatum and that they inhibit neuronal death in the nigrostriatal area in an MPTP‐induced PD model (Acuna‐Castroviejo, Coto‐Montes et al., 1997, Antolin, Mayo et al., 2002). Moreover, melatonin elevates the level of antioxidant enzymes, such as catalase and superoxide dismutase, in the nigrostriatal pathway in a 6‐OHDA‐induced PD animal model (Saravanan, Sindhu, & Mohanakumar, 2007). Another study has elicited similar results of the neuroprotective effect of melatonin in the mouse nigrostriatum of a 6‐OHDA induced PD animal model through inhibiting OH generation and preventing glutathione (GSH) reduction (Thomas & Mohanakumar, 2004). Moreover, melatonin clearly counteracts the reduction in mitochondrial oxidative phosphorylation enzyme (complex I) in the substantia nigra of a 6‐OHDA‐induced PD animal model (Dabbeni‐Sala, Di Santo, Franceschini, Skaper, & Giusti, 2001). In a maneb‐ and paraquat‐induced PD mice model, melatonin improves locomotor activity by reducing the rise in nigrostriatal dopaminergic degeneration and lipid peroxidation (Singhal, Srivastava, Patel, Jain, & Singh, 2011). By downregulating the oxidative stress effects, melatonin is shown to be a potent antioxidant, which can improve the prognosis in PD (Figure 3).

Toxic α‐synuclein is formed by α‐synuclein oligomerization, fibrillation, and finally by producing Lewy bodies, which mediates neurodegeneration cell death (Outeiro, Putcha et al., 2008). Melatonin has been shown to work as an anti‐assembly and to play a role in interfering with α‐synuclein toxic oligomer and α‐synuclein fibril, therefore reduceing α‐synuclein induced cytotoxicity (Ono, Mochizuki et al., 2012). This is consistent with the Western blot finding, which reported that melatonin inhibits arsenite‐induced apoptosis by reducing the accumulation of α‐synuclein in the rat brain (Lin, Fang, Chao, & Yang, 2007; Figure 3).

In humans, RT‐PCR of postmortem PD brains found reduced expression of MT1 and MT2 receptors in the amygdala and substantia nigra (Adi et al., 2010). Moreover, it has been reported that melatonin rhythm amplitude and 24‐hour plasma level are significantly reduced in PD patients compared to controls (Videnovic, Noble et al., 2014). These results reflect the involvement of the melatoninergic system in the pathophysiology of PD in humans. However, clinically, evidence has been accumulating that antagonizing the effect of a melatonin receptor by light therapy improves the motor outcome in PD patients (Paus, Schmitz‐Hubsch et al., 2007; Willis & Turner, 2007; Rutten, Vriend, van den Heuvel, Smit, Berendse, & van der Werf, 2012), consistent with some animal model studies (Willis, 2008; Cardinali et al., 2012). Further studies using external melatonin instead of light therapy are needed to better understand the role of melatonin in motor functions.

## 11 CONCLUSION

This review highlights the potential neuroprotective effect of melatonin in ischaemia, Alzheimer's disease, and Parkinson's disease. It is not only a widely known antioxidant, but also an anti‐excitotoxicity, anti‐ inflammatory, and anti‐misfolding molecule. Moreover, its ability to cross the blood–brain barrier and its short life with no significant side effects make melatonin a promising neuroprotector.

## CONFLICT OF INTEREST STATEMENT

The author has no conflicts of interest to declare.
